# Old Age

**Published:** 1865-01

**Authors:** 


Old Age.—There are few subjects of which the common impressions of men are more at fault than in respect to the proportion of our New England population which lives to old age. Ask the first man you meet, provided he be one who has never had occasion to examine the matter definitely and carefully, how many of our New England people (i. e., one in how many) live to be seventy years old; and very likely he will answer you, "not one in a hundred," or "not one in fifty." Death is a very common circumstance in this world, and so many die in infancy and childhood, that it comes to be regarded as something quite uncommon for a person to live to old age. Our Massachusetts statistics show that more than one in five of our native-born population live out their "three-score and ten." If any one doubts this statement, the facts to confirm it are abundantly set forth in our annual State registration reports.

But we wish for a moment to turn the attention of the reader to some considerations, which will help him to the truth, without recurrence to tables of life statistics. Let him go back to the families which he himself has known—if he be an old man, to those that started in life with him; or, if he be a young man, to those who were the contemporaries of his father and mother—and if he finds a family in which there were ten children, see if two of them at least did not live to be seventy years old. He may, it is true, hit upon a family where, through the prevalence of consumption, or some other form of hereditary disease, or through some strange combination of outward circumstances, all the children have died young. But the compensation for this will be, he can easily find another family, in which all, or almost all, have lived to great age. A case just reported in the papers, in connection with a death in the town of Hamilton, is exactly in point:

"In Hamilton, 30th ult., Benjamin Dodge, 74 years 7 months. He was the last of a family of thirteen children, whose father died at the age of 78, the mother at 94. One of the sons died at the age of 40, seven other sons lived to be from 74 to 90 years old, and the five daughters were all passed 60 at the time of their death."

In the family of the late Dr. Noah Webster were three sons and two daughters, no one of whom died until nearly 70, while the three sons lived to be 80 and upward. The long-lived families may therefore be set over against the short lived, and when the balance is struck, the case will be found as we have stated.

Take another class of facts. Harvard College has been in existence more than 220 years, and Yale College more than 160 years. The average age of young men when they graduate at Yale is between 21 and 22 years, and at Harvard a little less. Now it is no very uncommon circumstance to find that half the members of a class are alive at the end of fifty years from the time of graduation. The history of both colleges will show many instances of this kind. If we mistake not, the class of 1811 at Harvard, of which Hon. Edward Everett was a member, had nearly half its number alive at the time of the class meeting in 1861. And the men thus living must be at an average of more than 70 years. The general law, however, respecting our college graduates, will show a somewhat lower ratio than this. The living, at the end of fifty years from graduation, will be usually between one-third and one-half of the class.

The chances for living to old age in New England are good. There are few spots on the face of the earth better, in this respect. We sometimes talk about our bleak west and cruel east winds, as if they carried consumption and death into every household. We speak of our sudden and violent changes from heat to cold, and from cold to heat, as if everybody must go down under such sharp meteorological changes. But the truth is, on the broad scale, and in the long run, there is health rather than disease in these biting winds and decisive changes. The atmosphere is purified, and the system is toned up by the operation of these causes, which seem to us, at the time, "not joyous, but grievous."—*Boston Daily Advertiser.*

